# Applicability and Normative Data for an Arabic Matrix Sentence Test for Speech Recognition in Noise

**DOI:** 10.7759/cureus.77062

**Published:** 2025-01-07

**Authors:** Melanie A Zokoll, Michael Buschermöhle, Nadia Abdulhaq, Shaza Saleh, Nithreen Said, Khalid Abdulhadi, Fatma Sellami, Sabine Hochmuth, Birger Kollmeier

**Affiliations:** 1 Audiology, Hörzentrum Oldenburg gGmbH and Cluster of Excellence Hearing4all, Oldenburg, DEU; 2 Clinical Innovation Center for Medical Technology, KIZMO GmbH, Oldenburg, DEU; 3 Audiology, Cochlear Middle East and Africa, Dubai, ARE; 4 Audiology, King Abdullah Ear Specialist Center, King Saud University, Riyadh, SAU; 5 Otolaryngology, Ear Institute, University College London, London, GBR; 6 Audiovestibular Unit, Department of Otorhinolaryngology, Faculty of Medicine, Imam Abdulrahman Bin Faisal University, Dammam, SAU; 7 Audiology Unit, Department of Otorhinolaryngology, Ain Shams University, Cairo, EGY; 8 Audiology and Balance Center, Hamad Medical Corporation, Doha, QAT; 9 Department of Medical Physics and Acoustics and Cluster of Excellence Hearing4all, Carl von Ossietzky University of Oldenburg, Oldenburg, DEU; 10 Department of Otorhinolaryngology, Head and Neck Surgery, Carl von Ossietzky University, Oldenburg, DEU

**Keywords:** arabic speech recognition test, matrix sentence test, speech audiometry, speech in noise test, speech perception in noise, speech recognition threshold

## Abstract

Introduction: A matrix sentence test in Modern Standard Arabic (MSA) was developed with a multicenter approach to ensure high acceptability of the speech material. Normative values were obtained at four study sites located in the Persian Gulf region. Test characteristics were compared to other matrix sentence tests.

Methods: Forty-one native Arabic-speaking adults with normal hearing participated in two test development studies, 22 of whom participated in the normative study of the Arabic matrix sentence test. Speech intelligibility scores for individual words and test lists were obtained to optimize word materials and to verify equivalence of test lists, respectively. Normative speech recognition thresholds (SRTs) for adaptive test conduction were also obtained.

Results: Optimization of the test material with regard to homogeneous speech intelligibility of the individual words resulted in equivalent test lists with a mean SRT of -7.2 ± 0.2 dB signal-to-noise ratio (SNR) standard deviation (SD) and a slope at SRT of 13.3 ± 1.1%/dB. A mean SRT of -6.7 ± 1.1 dB SNR was obtained for adaptive SRT estimation. In addition, a training effect similar to matrix sentence tests in other languages was found.

Conclusion: With respect to homogeneity across individual words and test lists, as well as to normal-hearing SRT values and the slope of the discrimination function, the Arabic matrix sentence test achieves reproducible, efficient, and internationally comparable speech recognition data for native Arabic-speaking listeners.

## Introduction

Speech perception is one of the most important capacities of the human auditory system. Conversations generally occur in the presence of background noise. Hearing-impaired listeners often complain about problems with understanding speech, especially in noisy situations [1,2]. Suprathreshold deficiencies in the hearing process that may limit the utility of hearing devices in the rehabilitation process can only be assessed when individual auditory resolution and information extraction are tested at presentation levels above the individual absolute threshold [1,2]. Therefore, the diagnostics and rehabilitation of hearing loss now include speech audiometry in noise.

There are diverse test procedures for speech audiometry. In fact, each language and region has its variety of tests for assessing the speech intelligibility of individuals with hearing loss. Most speech intelligibility tests, including those in Arabic, use single, isolated words as stimuli presented in quiet. In 1956, Messouak developed the first test of this kind in the Maghrebian dialects [3], which was thought to be suitable for Algeria, Morocco, and Tunisia. Additional tests followed, some of which use literary Arabic with a phonetic balance of test lists, based on continuous text counts of consonants and vowels [3,4]. Ratcliff [5] developed the psychometrically equivalent bisyllabic words for speech recognition threshold testing in Arabic using digitally recorded, standardized bisyllabic Arabic words in the Palestinian/Jordanian dialects from different sources, such as daily newspapers, modern prose, second-language teaching, and children’s storybooks. Another test requires the detection of phonologically significant contrasts presented in varying phonetic contexts, such as the Arabic Speech Pattern Contrast (ArSPAC) test developed by Kishon-Rabin and Rosenhousea [6].

In addition to not being representative of everyday speech communication, the shortness of the utterances - especially for tests using monosyllabic words without a preparatory sentence - has some disadvantages. One disadvantage is that there might be a high rate of false responses in the absence of attention. Another disadvantage is that hearing aids with automatic gain control may have functional problems due to the brevity of the stimuli. Speech intelligibility tests that use complete sentences as speech material, compared to tests that use individual words, show several advantages [7]. Full sentences are more relevant to everyday life and allow for higher test reliability because more words can be tested per presentation and per unit of time. More words per presentation and per unit of time result in lower standard deviations (SDs) for speech intelligibility scores [8] and can lead to high precision in determining SRTs, e.g., for the unaided and aided case [7], which yields a precise assessment of the benefit from a hearing device in terms of an improvement in SNR by the hearing device.

Another critical issue is that conventional speech audiometric procedures are intended for assessing speech perception in quiet [9]. However, the threshold for understanding speech in quiet is highly correlated to the pure-tone audiogram (e.g., abstract: Brand and Kollmeier, Prediction of Speech Intelligibility in Quiet and in Noise on Basis of the Pure-Tone Audiogram (in German), 5th Annual Meeting of the German Society of Audiology (DGA); 2002, Zurich, Switzerland) and primarily assesses the attenuation component of a certain hearing loss [10]. In order to gather additional information about a patient’s hearing problem, suprathreshold speech perception performance should also be tested. This testing can reveal factors that are independent of the pure-tone audiogram. The factors are described as speech-hearing loss for people with speech perception problems in noisy conditions in spite of a normal pure-tone audiogram [11] or the distortion component of a hearing loss [12,10]. However, only a few speech intelligibility tests are specifically designed for use in noise. One example in the Arabic language is the Arabic version of the Hearing in Noise Test (HINT) [13]. The Arabic HINT consists of 28 phonemically balanced lists of 10 sentences each, spoken by a male speaker, and presented in multispeaker babble noise.

There are also potential drawbacks of speech tests using complete, meaningful sentences. For example, the complexity of the sentences or the speech rate may be too high for patients who are severely hearing-impaired or for patients who have cochlear implants [14]. Moreover, using meaningful everyday sentences allows patients to memorize the sentences, which might lead to biased repeated measurements.

A solution for these critical issues is using a limited set of words to create seemingly random but syntactically correct sentences with a fixed structure. This is the basic idea behind the matrix sentence tests first introduced by Hagerman [15]. In their review, Kollmeier and colleagues discussed the principles and applications of matrix sentence tests and compared different language versions of the test [16]. All sentences in matrix sentence tests have the same structure (e.g., in Arabic, the structure is verb, name, numeral, noun, and adjective, such as “يعطي فؤاد ثلاثة ألواح جميلة”). Test lists are generated by creating seemingly random sentences from an inventory, or base matrix, of 50 words (five positions per sentence and 10 words per position). Despite the seemingly random composition of the sentences, every sentence is syntactically correct. Up to 100,000 different sentences can be generated, although only a subset of the possible sentences is used in order to ensure test list equivalence. The seemingly random nature of the sentences makes it virtually impossible to memorize them. Matrix sentence tests are currently available for more than 20 different languages [17-20]. One of the advantages of matrix sentence tests is that the test format allows the comparison of speech audiometry across languages [16,21]. The number of available matrix sentence tests developed according to the respective standards is increasing [22], and therefore a growing number of patients can be tested in the same way for all languages, which creates a de facto standard for use in research and clinics.

With regard to Arabic-speaking countries, two other aspects of matrix tests might be interesting. First, due to its limitation in words, the matrix test might be relatively independent of dialects. Second, speech audiometry in a country’s minority languages is feasible by the multilingual versions of this test format. This is especially important because speech audiometry should be done in the native language of the patient; otherwise, a patient’s language deficits might be misinterpreted as hearing deficits [23]. This is supported by the closed-set test format of matrix sentence tests, which allows the patient to be tested in his or her native language using a touch screen for the response matrix (i.e., the closed-set response format) so that the test instructor does not need to understand the patient’s native language.

This paper describes the development and optimization, as well as the evaluation of the Arabic matrix sentence test. The test is intended both as a valid extension of speech audiometric tests available for the Arabic language and as a means to compare the performance of Arabic native listeners to international listeners with high comparability across languages. Because the Arabic language’s phonemic and syntactical structure differs from languages considered previously when constructing a matrix sentence test, the following research questions are addressed.

The first research question addresses the peculiarities of the Arabic language, which should be observed to create a test that can be used for the largest possible population. For this purpose, a joint project spanning researchers and normal-hearing listeners from several Arabic-speaking countries was initiated. However, an independent verification, i.e., whether reference values gathered with listeners from the Arabian Peninsula are valid for all dialectal regions of the Arabic language, is beyond the scope of this study. The second research question addresses the characteristics of the Arabic matrix sentence test, which can be designed, and, as a consequence, can be found via verification tests, to be comparable to those of other available matrix sentence tests, adhering to the respective standard procedures for development. Particular attention is given to ensuring the equivalence of the test lists, as well as on a small standard deviation between SRTs of listeners with normal hearing, obtained in the adaptive standard procedure [24]. The third research question addresses whether the Arabic matrix sentence test exhibits a similar training effect as described for matrix sentence tests in other languages [16].

This work was partially presented as a meeting abstract at the 11th Annual Middle East Otolaryngology Conference in Dubai, UAE, on April 20-22, 2014.

## Materials and methods

Development and optimization of the Arabic matrix sentence test

Speech Material

A significant characteristic of Arabic is diglossia [25], which refers to languages that have two levels or registers: an oral or colloquial dialects register and a literary or written register. Arabic is a pluricentric language. Diglossia is found in Arabic, earlier forms of Greek, and Swiss German [25]. Standard Arabic differs from colloquial Arabic in terms of its phonology, morphology, syntax, and lexicon. In order to be applicable to as many Arabic speakers as possible, the speech material of the Arabic matrix sentence test is based on Modern Standard Arabic (MSA), the written form of Arabic, which is used in official news broadcasts, school books, newspapers, and other written materials.

Analogous to the other matrix sentence tests, the base matrix of the Arabic matrix sentence test consists of 10 verbs, 10 names, 10 numerals, 10 nouns, and 10 adjectives (see Appendix). Each combination of words across word groups would result in grammatically correct sentences.

**Figure 1:**
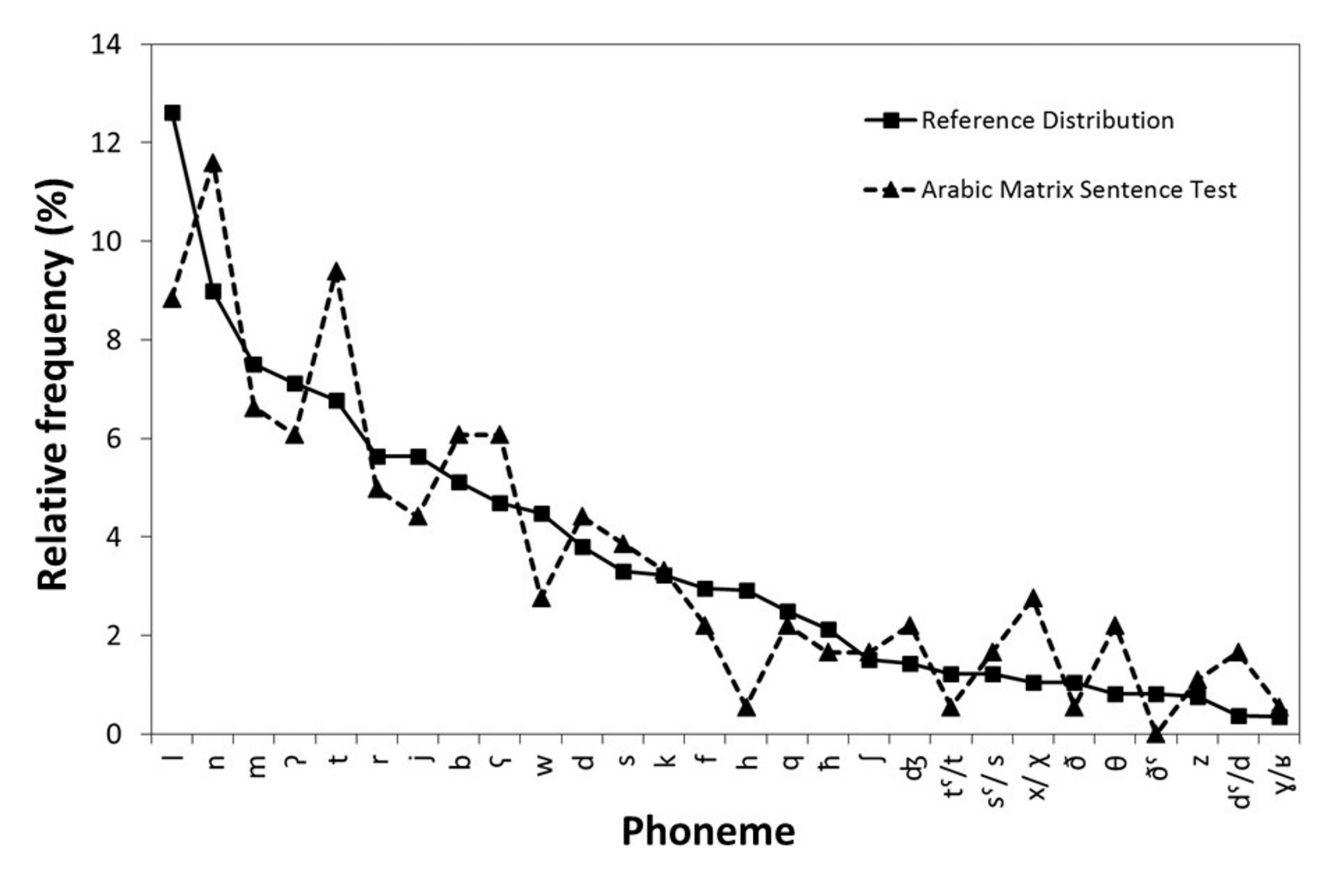
Phoneme distribution Phoneme distribution (relative frequency, in %) of the Arabic matrix sentence test (represented with triangles) compared to the phoneme distribution of MSA (represented with squares).

For the base matrix, words frequently occurring in MSA were selected, which could be suitable for testing school children. The nouns were chosen to represent common and tangible things. For numerals, numbers up to 10 and the words “few” and “many” were chosen. A balanced number of syllables within each word group was employed. As a final constraint, the speech material, as a whole, represented the phoneme distribution of MSA. The phoneme distribution published by Amayreh, Hamdan, and Fareh [26] was used as a reference. The reference and phoneme distribution of the Arabic matrix sentence test are displayed in Figure 1.

For the recordings, 100 sentences containing all possible combinations of two consecutive words of the base matrix were prepared to account for co-articulation effects between consecutive words [18]. The recordings were made in a sound-attenuated room with equipment meeting the requirements of the ISO-8253-3 standard. The speaker was a female TV news anchor from Syria who spoke clear and well-pronounced MSA. A consensus among the authors was reached that her MSA should be understandable to a large population in Arabic-speaking countries. She was instructed to keep the same speech effort, speech rate, speech level, and distance from the microphone at all times, as well as to use a natural intonation for main clauses. Five people, i.e., one Arabic linguist, one German linguist, one recording engineer, and two co-author researchers, listened to the recordings to identify artifacts or other recording errors and to monitor the speech effort, speech rate, and speech level. The raw recordings were high-pass filtered at 50 Hz in order to remove any existing humming noise. For the next processing step, the sound recordings were separated into individual sentences. To account for potential loudness differences, all sentences were then adjusted to the same RMS level. After that, the Arabic linguist selected the best, i.e., artifact-free and well-pronounced, version of each sentence. Finally, the 100 best sentences were cut into individual words, preserving the co-articulation to the following word at the end of the cut word. Test sentences were then resynthesized by quasi-randomly combining words of each word group with the appropriate transition to the following word. This way, new natural-sounding sentences were generated by concatenating words with matching co-articulations as proposed by Wagener and colleagues [18]. The sentences were combined to form fixed base-test lists of 10 sentences each. The base-test lists were prepared to ensure the sentences seemed purely random. In each base-test list, each of the words of the base matrix occurred only once, but in a different word combination, across base lists. The mean speech rate of the newly created sentences was 323 ± 18 syllables per minute.

Analogous to the matrix sentence tests in other languages, the noise for the Arabic matrix sentence test was created by a 30-fold superposition of the 300 newly combined sentences.

Listeners

The optimization measurements were performed with 19 normal-hearing listeners at three separate centers in Saudi Arabia and Qatar: 10 listeners at Dammam University in Saudi Arabia, four listeners at Hamad Medical Corporation in Qatar, and five listeners at King Faisal Specialist Hospital & Research Centre in Saudi Arabia. Normal hearing was verified by inspecting the listeners’ tone audiograms. Only listeners with thresholds ≤15 dB HL for octave frequencies from 125 Hz to 8 kHz were included in the study. The optimization measurements were conducted monaurally, and each listener’s best testing ear was based on the tone audiogram. If both ears were equal, the listener’s preferred ear was chosen. All participants were native speakers of Arabic. All listeners participated in the measurements voluntarily and gave their informed consent for storing and processing the data.

Procedure

The optimization measurements aimed to determine the psychometric function and, by that means, the slope and the SRT in the noise of each word in the test, i.e., all 10 realizations of the 50 words. The equipment used for the optimization measurements was a laptop PC with an Auritec USB audiometer (ear 3.0, Auritec, Hamburg, Germany) and audiometric headphones (AT 1350, Auritec, Hamburg, Germany). The software controlling the audiometer was the Oldenburg Measurement Applications (OMA, version 1.3) by Hörzentrum Oldenburg (Oldenburg, Germany). The speech material and noise material (when present) playback via headphones was monaural to the better ear. The equipment was calibrated to ensure all levels presented in this study are free-field equalized speech levels at the listeners’ ears, unless stated otherwise.

For the optimization measurements, the base-test lists of the Arabic matrix sentence test (each consisting of 10 sentences) were combined to form 10 different 30-item (30-sentence) test lists. Before the main optimization measurements, all listeners were trained by performing speech perception measurements with two 30-item test lists. The first training list was measured without noise at a constant level, providing high intelligibility. The second training list was performed with noise at a constant -4 dB SNR, which was expected to lead to an intelligibility of 100%. In all measurements with masking noise, the noise level was fixed at 65 dB, and the noise started 500 ms before, and ended 500 ms after, a sentence.

The 10 test lists were presented to each listener in a randomized order at 10 different SNRs (between -20 dB SNR and +2.5 dB SNR in 2.5 dB steps), so that each test list was measured one time at each SNR per listener. Word scoring was used as the scoring method. During the psychometric measurements, breaks were introduced after a test list measurement if the subjects were tired or had lost concentration. The psychometric function for each of the 500 words was determined by combining the data of all test subjects and fitting the following logistic function to the data:

 (1)$SI(SNR)=\frac{100}{1+e^{4 s50(SRT-SNR)}}$

SI(SNR) is the speech intelligibility in percentage at the SNR, and s50 is the function’s slope at the SRT.

Evaluation of the Arabic matrix sentence test

Speech Material

The evaluation measurements aim to verify that the 14 base-test lists that remained within the test material after optimization of the Arabic matrix sentence test (see Results section: *Development and optimization of the matrix sentence test*) are equivalent with respect to speech intelligibility in noise. The evaluation measurements also provide reference values for further applications, such as the SRT and the range of normal performance for speech in noise (presented monaurally via headphones), as well as orientation for the training (training effect).

Listeners

The evaluation data were gathered at four separate centers: Dammam University (N = 10), King Faisal Specialist Hospital & Research Centre (N = 9), Cochlear Middle East FZ-LLC in Dubai (N = 2), and Hamad Medical Center (N = 1). Normal hearing participants were recruited in the same manner as for the optimization measurements. None of the participants contributed to the previous optimization measurements.

Procedure

The remaining 14 10-item base lists were combined into seven 20-item test lists for the evaluation measurements. All measurements during the evaluation were performed monaurally using each listener’s better ear. The same technical equipment was used as for the optimization. To investigate the training effect for the Arabic matrix sentence test, all listeners initially participated in seven consecutive test lists with adaptively changing SNR to obtain SRTs in noise. The applied adaptive procedure kept the noise level constant at 65 dB and varied the speech level according to the listener’s responses. The first sentence is presented with an SNR of 0 dB. If the subject correctly repeated more than 50% of the presented words, the speech level of the following presentation was reduced. If the subject repeated fewer than 50% of the words correctly, the next presentation’s speech level was increased. The step sizes varied between 7.5 dB for the first trials and 0.25 dB SNR for later trials, depending on the distance to the targeted intelligibility, i.e., 50%, and the number of preceding turning points within the measurement. A maximum likelihood estimator determined the SRT. Brand and Kollmeier provide more details on the adaptive procedure [24].

In the evaluation, 21 measurements at a fixed SNR were performed (all seven 20-item lists at each of three SNRs: -6, -8, -10 dB SNR; 65 dB noise level). SNRs were selected to optimally access the slope of the test-list-specific speech intelligibility function, i.e., to obtain approximately 20%, 50%, and 80% intelligibility [24]. The order of the test lists and the SNRs were randomized. Between the seventh and eighth measurements at a fixed SNR, eight of the listeners had a break lasting between one and seven days. Fourteen listeners were tested on only one day. Three of those listeners completed only seven of 21 measurements at fixed SNRs. The scoring method for both measurement profiles was word scoring.

Data Analysis

The initial SRT measurements were used to investigate the training effect of the Arabic matrix sentence test. SRTs for all 22 listeners and the seven consecutive test lists were included in the analysis. Because the SRTs were not normally distributed, a one-way repeated measures ANOVA on ranks (Friedman method, SigmaPlot) was performed on the SRTs with measurement numbers as the within-subjects factor. For additional pairwise multiple comparisons, Tukey tests were employed. The measurements at fixed SNRs were used to calculate test list-specific speech intelligibility functions using Equation 1 to assess the test list equivalence based on the parameters describing the functions. For this purpose, data obtained with fixed SNR were combined. Data obtained for 20-item test lists were sorted according to their respective 10-item base-test list, and intelligibility was calculated for each base-test list, each listener, and each SNR. Test list equivalence was verified through statistical analysis. For this purpose, a two-way RM ANOVA was evaluated on the intelligibility with the “base-test list” and “SNR” as factors. In addition, all pairwise multiple comparison procedures (Holm-Sidak method) were used for further analysis of significant main effects. For all these statistical analyses, a p-value of 0.05 or less was considered to be significant. To obtain the parameters describing the speech intelligibility functions for both the individual base-test lists and the individual listeners, psychometric functions were fitted to the data. To obtain test list-specific intelligibility functions, a logistic function (Equation 1) was fitted to the combined intelligibility data obtained, across subjects, for a specific test list. By these means, the SRT and slope describing the intelligibility function for each base-test list can be obtained. To obtain listener-specific intelligibility functions, the logistic function was fitted to the combined intelligibility data, across base-test lists, for a particular listener.

The Research Ethics Committee of the University of Oldenburg issued approval (no. Drs. 21/20/2013).

## Results

Development and optimization of the Arabic matrix sentence test

**Figure 2:**
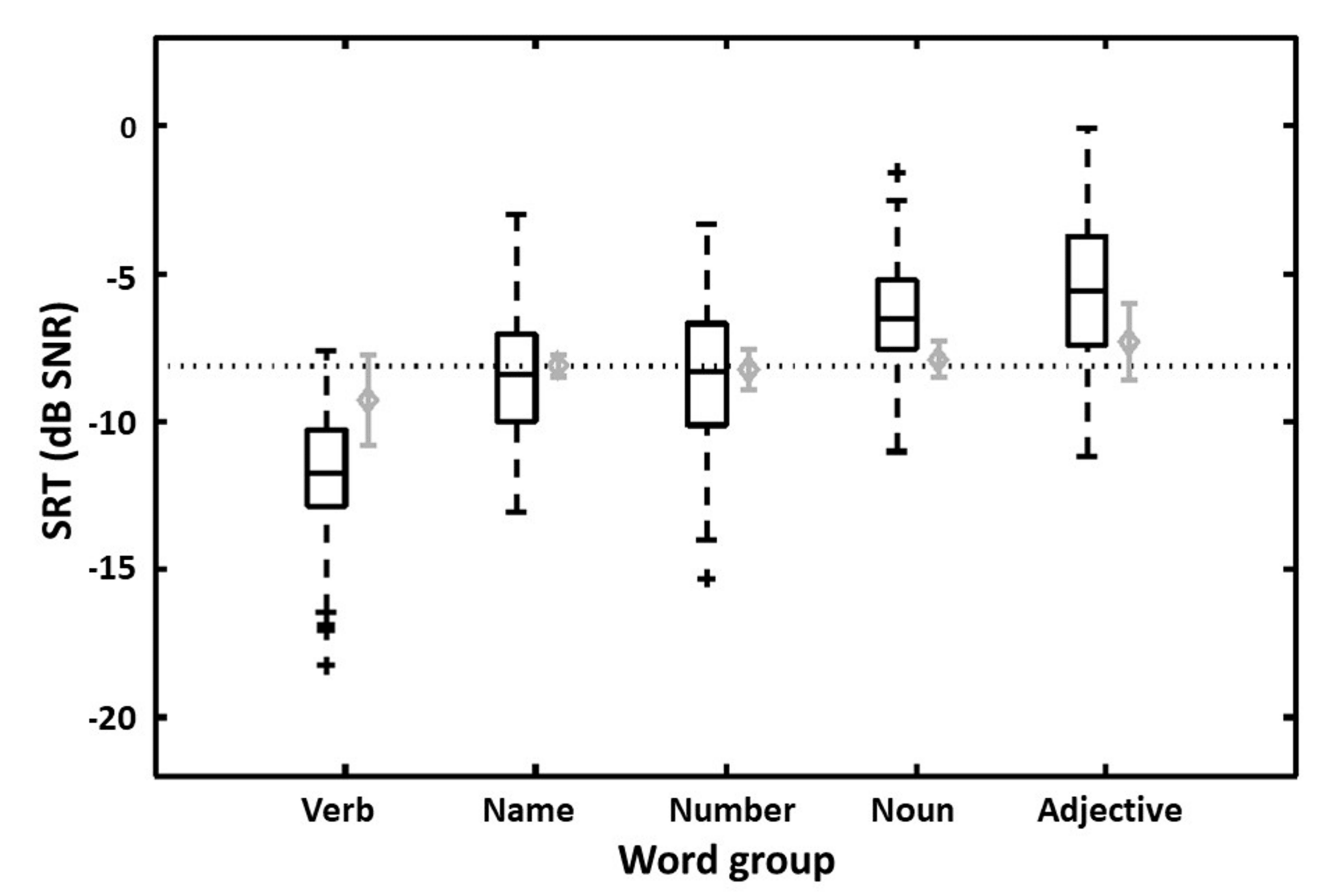
Word-specific speech recognition thresholds (SRTs) Word-specific SRTs resulting from the optimization measurements for the different word positions in the sentence. SRT is the signal-to-noise ratio resulting in 50% speech intelligibility (in dB SNR). Represented in black: Mean, standard deviation (as box size), and 95% range (whiskers), plus extreme cases (crosses) for the word-specific SRTs (in dB SNR). Represented in gray: Predicted word-specific SRTs (mean and standard deviation, in dB SNR) after level adjustments.

The approach to test speech intelligibility in noise as a basis for optimization yielded an average SRT in the noise of all words of -8.1 ± 3.0 dB SNR (SD). The word-specific SRTs differed slightly between word groups, as depicted in Figure 2. In the second step, each word was then adjusted in level to match the average SRT in the noise of all words, with the assumption that this procedure also changes intelligibility to the same extent. In this way, words that were more intelligible than the average were lowered in level, and less-intelligible words were increased in level in accordance with the general optimization procedure of matrix sentence tests [16]. These level adjustments were limited to ±3 dB to preserve the natural intonation of the sentences, which a native speaker additionally confirmed after the level adjustments. By adjusting the levels of the words, the expected word-specific SRTs could be brought close to the average SRT, resulting overall in an expected SRT of -8.1 ± 1.2 dB SNR (SD).

In an additional optimization step, 15 10-item base-test lists could be identified that contained no word with an expected SRT that deviated more than ±3 dB from the target SRT of -8.1 dB SNR. Of those 15 test lists, one test list with an average word-specific slope of less than 5%/dB was discarded, and therefore 14 10-item test lists remained in the speech material. These remaining test lists had an expected mean word-specific SRT of -8.1 ± 1.0 dB SNR (SD) and a mean word-specific slope of 15.9 ± 6.6 %/dB (SD). The final sentences within these test lists, created by re-concatenating the level-adjusted words, were judged to sound natural by a native speaker.

Evaluation of the Arabic matrix sentence test

Training Effect

**Figure 3:**
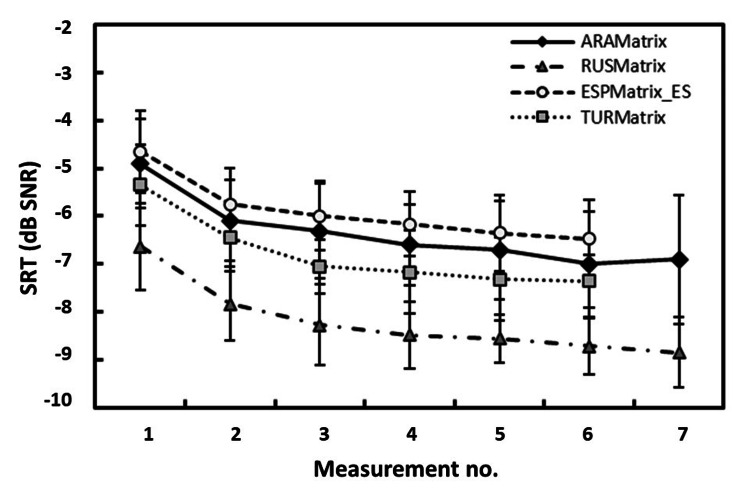
Training effect Training effect, i.e., speech recognition thresholds (SRTs, in dB SNR) as a function of measurement sequence number with 20-item test lists using the adaptive procedure (means and standard deviations). Shown here are the curves for the Arabic matrix sentence test (ARAMatrix) and comparable training effect curves for matrix sentence tests in Russian (dot-dashed line, RUSMatrix), Spanish (dashed line, ESPMatrix_ES), and Turkish (dotted line, TURMatrix).

For the first through the seventh 20-item test lists measured, the listeners improved on average by 2.0 dB SNR in SRT (Figure 3). The major contribution to the training effect was found within the first two measurements (approximately 1.2 dB SNR). The difference between the first and the third measurements was 1.4 dB SNR on average. After the third measurement, the improvement was marginal. The difference between the third and seventh measurements was 0.6 dB SNR, leading to a mean SRT of -6.7 ± 1.1 dB SNR (SD).

When comparing the SRTs obtained at the two sites with an adequate number of participants (two test sites; one with nine participants, and one with 10 participants), no significant differences could be found; therefore, data for all four sites were pooled to investigate the training effect. The RM ANOVA on ranks confirmed a significant main effect for the factor “measurement number” (χ2 = 62.043, p < 0.001). A follow-up analysis using pairwise multiple comparison procedures revealed that SRTs for the first measurements were significantly worse than for the third, fourth, fifth, sixth, and seventh measurements (all p < 0.05), but not for the second. The second measurement was significantly worse than the sixth and seventh measurements (both p < 0.05). Note, however, that the size of the training effect (approximately 1.4 dB within the two initial test lists) is small, i.e., lower than the resolution of most speech tests with the same number of test items. Its significance is only detectable due to the high accuracy and reproducibility of the matrix test results.

For comparison, the training effect curves for the matrix sentence test in Russian, Spanish, and Turkish are plotted in Figure 3, and they show the same dependencies. This indicates a high comparability of the Arabic matrix sentence test with respect to the training effect to matrix sentence tests in other languages. Hence, it can be assumed that the above-mentioned matrix tests show a similar training effect in naive listeners that can only be observed if a similar accuracy in estimating the SRT is achieved.

Test List Equivalence

**Figure 4:**
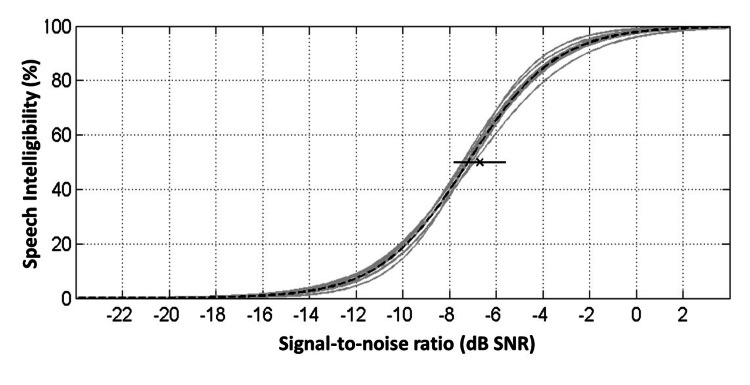
Psychometric functions Psychometric functions of the individual 10-item test lists (gray solid lines) and average psychometric function (black dashed line). Functions show speech intelligibility (in %) as a function of the signal-to-noise ratio (in dB SNR). In addition, the mean and standard deviation of the adaptively determined speech recognition thresholds (SRTs, in dB SNR) are depicted as cross and horizontal line.

Table 1 summarizes the mean intelligibility for the different SNRs, as well as the SRTs and slopes describing the functions calculated for the 14 10-item base-test lists. The initially selected SNRs (-6, -8, and -10 dB) only partially resulted in the targeted intelligibility of 80%, 50%, and 20%, respectively. The mean SRT and slope of the 14 base-test lists were -7.2 ± 0.2 dB SNR (SD) and 13.3 ± 1.1%/dB (SD), respectively. The mean SRT and slope of the listeners (not shown in Table 1) were -7.2 ± 1.2 dB SNR (SD) and 15.1 ± 3.1%/dB (SD), respectively. This indicates that the subject-specific variability in SRT leads to a slightly smaller slope across subjects and lists than the subject-specific slope. The psychometric functions of all 14 base-test lists are plotted in Figure 4.

**Table 1:** Speech intelligibility Speech intelligibility (SI, in %) per test list from 22 listeners at three signal-to-noise rations (-6, -8, and -10 dB SNR). The parameters speech recognition threshold (SRT, in dB SNR), i.e., the signal-to-noise-ratio resulting in 50% speech intelligibility, and slope at SRT (in %/dB) for each test list were determined by fitting a logistic model function (Equation 1) to the individual SIs. Mean and standard deviation across test lists are also shown.

	SI [%] at			Calculated	
10-item test list no.	-6 dB SNR	-8 dB SNR	-10 dB SNR	SRT (dB SNR)	Slope (%/dB)
1	65.8	36.8	21.0	-7.2	12.6
2	69.3	44.6	18.6	-7.4	13.8
3	60.9	34.6	16.5	-6.8	13.0
4	64.8	43.0	19.6	-7.3	12.5
5	65.6	41.4	17.5	-7.2	13.6
6	61.0	37.3	20.3	-6.9	11.4
7	64.6	39.3	15.4	-7.1	13.8
8	71.6	43.5	18.7	-7.6	15.0
9	68.5	38.6	14.3	-7.2	16.0
10	65.1	42.6	19.6	-7.3	12.6
11	66.3	39.0	19.1	-7.2	13.3
12	66.5	43.5	19.5	-7.4	13.1
13	62.5	40.0	15.4	-7.0	13.6
14	64.0	44.1	19.5	-7.3	12.3
Mean	65.5	40.6	18.2	-7.2	13.3
SD	3.0	3.1	2.1	0.2	1.1

Furthermore, test list equivalence was verified through statistical analysis. The two-way RM ANOVA was evaluated on the intelligibility. The ANOVA excluded subjects with incomplete data. Therefore, data from only 19 of 22 listeners were included in the analyses. For both factors, i.e., base-test lists and SNR, significant main effects were found (F(13,234) = 4.789 for base-test lists and F(2,36) = 589.565 for SNR, both p < 0.001). There was no significant interaction of the base-test list with SNR. Pairwise comparisons showed that test list number 3 differed significantly from six other test lists (test lists numbers 2, 4, 8, 10, 12, and 14, all p < 0.05). Test list number 8 differed significantly from test list number 13 (p < 0.05). Furthermore, test list number 2 tended to differ from test list number 7 (p = 0.048). In conclusion, the particularly conspicuous test lists numbers 3 and 8 were excluded from the final speech recognition test material. The final 10-item test lists were combined to form 24 new 20-item and 20 new 30-item test lists constituting the final Arabic matrix sentence test.

## Discussion

The Arabic matrix sentence test presents a new speech audiometric sentence-in-noise test for Arabic speakers that has been constructed to fulfill the following requirements.

The test will be appropriate for a large population in Arabic-speaking countries. This was achieved by selecting a base matrix with a carefully selected choice of words from MSA - a pluricentric auxiliary language used in formal education - utilizing a speaker who employs standard pronunciation, as determined within a multisite consortium representing several Arabic-speaking countries. The multisite verification measurements of the test in the Persian Gulf region yielded no indication of any objections against the speaker’s pronunciation and no significant effect of the test site.

By adhering to the ICRA test development recommendations [22], the test properties are comparable to matrix sentence tests available in other languages [16]. The test exhibits a recording speech rate of 323 ± 18 syllables per minute, an average SRT of -6.7 ± 1.1 dB SNR (SD) across subjects using the adaptive procedure (or -7.2 dB for the constant-level procedure), and a standard deviation of 0.2 dB across test lists. These results align with other matrix sentence tests, which typically have SRTs in the range of -10 dB SNR to -6 dB SNR.

The test yields a high precision for estimating the SRT due to a steep slope of 13.3 ± 1.1%/dB (SD, variability across test lists). Furthermore, this value is in the range of 10%/dB and 17%/dB for matrix tests in other languages [16]. If the variability of SRTs for individual listeners is taken out by computing the average listener-specific slope, an even higher value of 15.1 ± 3.1%/dB (SD) is found. It is important to note that the variability of the individual percent score is governed by the binomial distribution, which yields a particular variance for a given target probability and a number of trials. Because the slope converts this variability of the individual percentage-correct score into the SRT domain, a very low variability of SRT results from sufficiently steep slopes. Hence, a high precision of the Arabic matrix sentence test is achieved. The Arabic matrix sentence tests exhibit a significant training effect (Figure 3), which compares well with the tests from other languages [17,19-20]. As a clinical consequence, training with two test lists of 20 sentences each is necessary prior to the actual measurement for the Arabic matrix sentence test.

With respect to the representativeness of the Arabic language and research question 1 (peculiarities of the Arabic language to be observed), it should be noted that several design decisions of the Arabic matrix test were performed in advance by the team of authors. These decisions were partially based on evidence and common knowledge of the contemporary Arabic language (and its varieties) and were influenced by the demand to follow the recommendations by Akeroyd and colleagues [22] as closely as possible. Hence, the test was recorded with a professional speaker of MSA, and special recording and sound-processing techniques ensured that all sentences sounded very natural despite word concatenation. The MSA literary version was employed instead of a spoken dialect. In general, using MSA and its literary version is an appropriate decision. Special care was taken in the selection of words to make the test suitable for school children; however, this aspect was not validated further in this study. It needs to be determined in more depth whether using the MSA would result in different SRTs when used in the different dialectal regions of the Arabic-speaking countries. Testing this aspect in more detail was beyond the scope of the current study. However, for the Spanish matrix sentence test [17] and the American English matrix sentence test (unpublished data), effects due to dialects were marginal, (e.g., below 1 dB for the Spanish matrix sentence test) [17]. Dialect differences did not result in different reference SRTs for normal-hearing listeners. All subjects came from the same region, but some variation in the dialects between the subjects may have existed. However, all could undertake, understand, and complete the matrix sentence test. In addition, the standard deviations for SRTs in the current study are small (1.1 dB). Hence, we expect no systematic difference across regions that differ in their respective Arabic dialect and accent. However, this needs to be investigated in further studies.

With respect to comparability to other languages and tests and research question 2 (verification of comparability), it should be noted that much care was taken to optimize the recorded speech materials to make the test items as homogenous as possible with respect to their respective intelligibility. To this end, slight level corrections (up to 3 dB) were performed to equalize the word-specific discrimination function. Moreover, deviant words with regard to intelligibility and slope were removed from the material. This step is required to maintain the equivalence of the various test lists compiled from the recorded speech material and to ascertain that the slope of the resulting discrimination function is as steep as possible. Following the model by Kollmeier from 1990 [16], the smaller the variability across test items, the steeper the simultaneous transition from “not understood” to “perfectly understood” for the complete list, thus resulting in a steep discrimination function.

The evaluation measurements with new subjects verified the test’s properties by analyzing normal-hearing SRT and equivalence in intelligibility on the level of the individual base-test lists. After optimization and evaluation, 24 test lists of 20 sentences each and 20 test lists of 30 sentences each were established. A mean SRT of -6.7 dB SNR was obtained for adaptive SRT estimation with the Arabic matrix sentence test. This value differs slightly from the value obtained for measurements at fixed SNRs (-7.2 dB SNR). However, this difference between adaptive measurements and those at fixed SNRs also was observed in other studies [19,20] and most likely reflects the different estimation methods and training status of the listeners, i.e., the measurements at fixed SNR were conducted later in the session than the adaptive measurements [27]. For the Arabic HINT, Essawy and colleagues found an SRT in noise of -10.4 dB SNR, for signal and noise presented from the front [13]. This SRT in noise value is lower than what was found for our test. Differences between the two test materials can be attributed to several effects. Among them, the effect of the speaker might be the most striking. Hochmuth and colleagues found differences of up to 6 dB due to the speaker, as some speakers are more intelligible than others [28]. To a smaller extent, differences can be due to language characteristics. Some languages might exhibit a general language-specific intelligibility advantage (e.g., Russian over Spanish) [28]. Furthermore, HINT employs everyday sentences, which have a certain redundancy that might add to the difference between the two tests.

With respect to precision in estimating the SRT, it should be noted that a high slope of the discrimination function was both targeted in the design of the test and its optimization procedure, which was verified in the subsequent evaluation of test properties. Consequently, the mean slope of the Arabic matrix sentence test (based on the list-specific intelligibility functions), estimated by fitting Equation 1 to the data of the evaluation measurements at fixed SNRs, exhibits a steepness of 13.3 ± 1.1%/dB (SD). The mean slope value is comparable to other speech tests using sentences and the matrix test format [16]. Even though the value was below expectations based on the optimization measurements where a word-specific slope of 15.9%/dB had been found, the average subject-specific slopes (15.1 ± 3.1%/dB (SD)) better reflect the expectations because the respective individual bias of the normal-hearing subjects has been removed.

The slope usually found for monosyllabic words and also described for the Arabic speech material of this kind (5%/dB) [4] is rather shallow in comparison to the slope of the speech intelligibility function for the Arabic matrix test obtained here. A detailed discussion of measurement accuracy at different points of the psychometric functions and, subsequently, the dominant role of the maximum slope of the psychometric function for efficiently determining the SRT is given by Brand and Kollmeier [24]. Hence, the finding of a steeper slope (i.e., a slope value three times higher than that for the monosyllabic word test) provides evidence that the Arabic matrix sentence test is more accurate in estimating thresholds than these monosyllabic word tests. As a practical consequence, the Arabic matrix sentence test should be preferred in rehabilitative audiology whenever determining an SRT is important.

With respect to the training effect and research question 3 (comparability of the training effect), it should be noted that the training curve is very similar to those obtained for the Turkish, Russian, and Spanish matrix sentence tests (Figure 3) and other language versions of the matrix sentence test [16-20]. As noted by Warzybok and colleagues, this training effect is very similar for the open-set version of the test (i.e., the test subject responds by repeating that sentence she or he has understood) and the closed-set version (i.e., the subject responds by selecting those words from the 5 x 10-word matrix that she or he has understood) [19]. Hence, the training effect is not influenced by previous knowledge of the subjects about the words to be expected but rather resembles a perceptual learning task where the subject successively learns to exploit more cues. The similarity of training effects across languages strongly suggests common underlying factors and processes, supporting the conclusion that data obtained with matrix sentence tests from different countries can be combined and compared. A more detailed analysis of the mechanisms behind the training of the matrix sentence test is provided by Schlüter and colleagues [27].

In addition, it is important to note that the training effect observable for the matrix sentence test is not a specific property of the matrix sentence test. Yund and Woods [29] reported such effects for the HINT [14] and Quick Speech in Noise (QuickSIN) (handbook: Etymōtic Research: QuickSIN Speech-in-Noise Test, 2001, Etymotic Research, Inc.) tests, both of which consist of everyday sentences. However, for everyday sentences, Yund and Woods could differentiate between a small effect (about 0.3 dB) attributed to procedural learning, more dominant in the first session, and an effect due to content learning that was approximately 2.7 dB when the same lists were repeated within and across sessions [29]. This differentiation is not possible for a closed test like the matrix sentence test, because all possible words are present in every single list. Both procedural learning and content learning take place in parallel and might also be “completed” faster than the time it would take for everyday sentences. The size of the combined effect, however, is about the same for both tests. Similar training effects are well known from psychoacoustic tasks, e.g., signal detection in noise [30]. A training effect can only be detected if the measurement error achieved within a limited number of trials is smaller than the training effect across these trials. Speech tests with a small test efficiency (e.g., single-word tests with a shallow discrimination function noted above) will therefore be unable to detect this effect because their statistical test variability is higher than the effect to be observed.

## Conclusions

The research questions outlined in the Introduction can be answered as follows. Regarding the first research question, peculiarities of the Arabic language, a multisite approach, and the selection of a professional MSA speaker resulted in acceptable speech material at all sites, thus spanning a large geographical region and population of potential users. Regarding the second research question, characteristics of the Arabic matrix sentence test and comparability to matrix sentence tests in other languages are given. The Arabic matrix sentence test is equivalent with respect to its main characteristics to other matrix sentence tests. Hence, the Arabic matrix test results can be used in multisite, multilanguage studies. Furthermore, regarding the third research question, the Arabic matrix sentence test shows a training effect within the first two to three test lists performed with naive listeners. For clinical applications, it is therefore recommended to train the naive subject with two test lists before data collection begins. The new Arabic matrix sentence test promises to fulfill the need to obtain reproducible, efficient, and internationally comparable speech recognition data for native Arabic-speaking listeners in clinics, in research, and for numerous hearing rehabilitation purposes.

## Appendices

**Table 2:** Base matrix of the Arabic matrix sentence test A random sample sentence is written in bold letters. Each base sentence is translated into English in the far-right column.

Adjective	Noun	Numeral	Name	Verb	Sentence in English
جديدة (new)	كتب (books)	عدة (many)	علي (Ali)	يريد (wants)	Ali wants many new books.
بنية (brown)	اطباق (plates)	خمسة (five)	نبيل (Nabil)	اشترى (bought)	Nabil bought five brown plates.
صغيرة (small)	كراسي (chairs)	عشرة (ten)	زين (Zain)	يصنع (makes)	Zain makes ten small chairs.
كبيرة (large)	كؤوس (cups)	أربعة (four)	ناجي (Naji)	ربح (won)	Naji won four large cups.
خفيفة (light)	قمصان (shirts)	ستة (six)	عمر (Omar)	يفضل (prefers)	Omar prefers six light shirts.
حمراء (red)	خواتم (rings)	سبعة (seven)	هاشم (Hisham)	لون (colored)	Hisham colored seven red rings.
قديمة (old)	بيوت (houses)	بضع (few)	وائل (Wail)	نال (got)	Wail got a few old houses.
ثمينة (precious)	سكاكين (knives)	تسعة (nine)	بلال (Bilal)	يأخذ (takes)	Bilal takes nine precious knives.
زرقاء (blue)	اعلام (flags)	ثمانية (eight)	أمين (Amin)	أخرج (removes)	Amin removes eight blue flags.
جميلة (beautiful)	ألواح (boards)	ثلاثة (three)	فؤاد (Fuad)	يعطي (gives)	Fuad gives three beautiful boards.
